# Evidence of the Trade-Off between Starvation and Predation Risks in Ducks

**DOI:** 10.1371/journal.pone.0022352

**Published:** 2011-07-18

**Authors:** Cédric Zimmer, Mathieu Boos, Nicolas Poulin, Andrew Gosler, Odile Petit, Jean-Patrice Robin

**Affiliations:** 1 Université de Strasbourg, IPHC, Strasbourg, France; 2 CNRS, UMR 7178, Strasbourg, France; 3 Research Agency in Applied Ecology Naturaconst@, Wilshausen, France; 4 Edward Grey Institute of Field Ornithology, Oxford University, Oxford, England; University of Bristol, United Kingdom

## Abstract

The theory of trade-off between starvation and predation risks predicts a decrease in body mass in order to improve flight performance when facing high predation risk. To date, this trade-off has mainly been validated in passerines, birds that store limited body reserves for short-term use. In the largest avian species in which the trade-off has been investigated (the mallard, *Anas platyrhynchos*), the slope of the relationship between mass and flight performance was steeper in proportion to lean body mass than in passerines. In order to verify whether the same case can be applied to other birds with large body reserves, we analyzed the response to this trade-off in two other duck species, the common teal (*Anas crecca*) and the tufted duck (*Aythya fuligula*). Predation risk was simulated by disturbing birds. Ducks within disturbed groups were compared to non-disturbed control birds. In disturbed groups, both species showed a much greater decrease in food intake and body mass during the period of simulated high risk than those observed in the control group. This loss of body mass allows reaching a more favourable wing loading and increases power for flight, hence enhancing flight performances and reducing predation risk. Moreover, body mass loss and power margin gain in both species were higher than in passerines, as observed in mallards. Our results suggest that the starvation-predation risk trade-off is one of the major life history traits underlying body mass adjustments, and these findings can be generalized to all birds facing predation. Additionally, the response magnitude seems to be influenced by the strategy of body reserve management.

## Introduction

The competition between two or more processes for the allocation of limited resources generally results in a trade-off that underlies different life-history traits [1]. One important trade-off occurs for species acquiring food while avoiding predation [2], [3]. Animals have to build up or maintain body fuel reserves which are an important buffer against starvation, especially when harsh winter weather conditions involve unpredictable food availability and energy requirements [4]. However, it is surprising to see that birds often maintain their level of body reserves below the maximum threshold [5]. Assuming that maintaining a high level of body fuel, i.e. a high body mass, also incurs a significant cost in terms of enhanced mortality risk due to predation vulnerability [5], the amount of body reserves that a bird carries has generally been viewed as a trade-off between the risk of starvation and the risk of predation [6], [7]. Body mass adjustment is considered to be the consequence of this trade-off.

In this context, the mass-dependent predation risk theory predicts that if the probability of an individual being caught by a predator depends on its body mass, its weight should be maintained at an appropriate level to balance predation risk against the risk of starvation [6], [8]–[10]. Such body mass adjustment has the advantage of improving flight performance and reducing the associated metabolic demands. It also results in a lower investment in foraging time and less exposure to predation [7], [10], [11]. A high body mass is correlated with high wing loading and a greater cost of flight. These two factors could impair flight performance, particularly during take-off, due to a smaller angle of ascent and a lower speed [10], [12]–[18]. Conversely, birds have to maintain a level of body reserves which is high enough to limit the risk of starvation [6], [10]. It has generally been assumed that this strategy would lead animals to carry greater body reserves when starvation risk is high and vice versa [6], [13]. Nevertheless, empirical data on the starvation-predation risk trade-off that illustrates a decrease in body mass when individuals are under higher predation risks mainly originate from studies on small passerine birds [2], [19]–[26]. Furthermore, experimental studies have demonstrated that when predation risk was increased or when predator attacks were simulated by chasing the birds, food consumption decreased in order to adjust body mass [19], [21], [23]. This body mass adjustment improves take-off performance because the available power for flying increases when body mass declines [27] and this ultimately maximizes survival.

To our knowledge, apart from the afore-mentioned studies of passerines the only other species studied in relation to the starvation-predation risk trade-off are the redshank (*Tringa totanus*) [28], a larger species, the mallard (*Anas platyrhynchos*) [29] and one non-bird species, the harbour porpoise (*Phocoena phocoena*) [2]. In the two last species, it has been shown that body mass or body reserves were linked to predation risk. In mallards, the relative body mass decrease was twice as high as in passerines [29] and it was hypothesized that this was due to a difference in body mass and the amount of body reserves of each species: whereas passerines build up body reserves during the day and use them during the following night for energetic purposes [4], [30] mallards store more body reserves than required immediately in order to cope with possible future periods of cold spells [31]–[33]. Furthermore, large birds have higher body reserves and a lower metabolism per unit body mass than small species which have a higher surface/volume ratio [34]. Thus, large birds can sustain greater body mass variations than small ones, even in proportion to lean mass, without dramatically increasing their starvation risk. Moreover, this is consistent with the idea that greater body mass loss allows a greater power margin gain in large birds than in passerines. The power margin is defined as the ratio between power available and power required for flight. It therefore appears that the magnitude of the response to increased predation risk depends on species size, with a higher body mass loss in large birds than in small ones due to the difference in the amount of body reserves stored [29].

The present study was carried out on one small and one medium-sized duck species, the common teal (*Anas crecca*) and the tufted duck (*Aythya fuligula*) respectively. Predation risk was artificially increased in order to confirm that the starvation-predation risk trade-off applied in large birds. These two species were chosen as their exposure to predation is similar to that of other duck species sharing the same habitat (see [29]). Moreover, teals and mallards have similar body reserve dynamics throughout their biological cycle [35], and while diving tufted ducks show a similar body weight variation to dabbling ducks (i.e. mallards and teals) during winter [36], in terms of size they are intermediate between teals and mallards. They also differ from passerines in both size and body fuel storage strategy, with mass variations that are low on a daily basis but are seasonally high [4], [30], [35], [36]. We predicted that the extent of body mass loss in these two duck species should be greater than in passerines: although they need to improve their escape performance, the response should be approximately the same as that observed in the mallard, because the three species have the same relative amount of body reserves [37], [38].

## Methods

### Ethics Statement

This work was performed with governmental authorizations delivered by the Préfecture du Bas-Rhin (Strasbourg, France) to conduct experiments on ducks numbers 67-99 and 67–285, and was approved by the Direction Départementale des Services Vétérinaires du Bas-Rhin (Strasbourg, France). The experiment complied with the “Principles of Animal Care” publication No. 86–23, revised 1985 of the National Institute of Health, and with current legislation (L87–848) on animal experimentation in France. After the study, ducks were released in the field under the control of the “Office National de la Chasse et de la Faune Sauvage” and with the authorization of the “Direction Départementale de l'Agriculture et de la Forêt du Bas-Rhin”.

### Animals and experimental conditions

The study was conducted on 42 common teals (21 females and 21 males) from the Fauna Leroy rearing centre (Westvleteren/Belgium) and 28 tufted ducks (14 females and 14 males) from the “Les Canards de Mormal” rearing centre (Jolimetz/France). Groups of 14 individuals (7 males and 7 females) were constituted in both species: three groups in teals and two groups in tufted ducks. Only two groups could be studied in the latter species. This was due to a limited supply of individuals, which was insufficient for three groups to be created within the same season. Each group of 14 individuals was maintained in an outdoor tunnel aviary of 100 m^2^ (20×5×2.5 m). Each aviary contained a 4 m^2^ pool (0.60 m depth) containing clear running water which was positioned at the same location in each tunnel. Birds were subjected to natural photoperiod and ambient temperature. A species-specific balanced commercial diet (Standard duck food 7751, Sanders Corporation; Teurlings premium duck food) was provided *ad libitum.* The food was provided in feeders placed on 2×2 m tarpaulins to account for food spillage. The aviaries were located close to the laboratory, and were protected against predators within an electric enclosure and visually separated by opaque barriers. A two-month period of acclimation to the aviaries was applied for both species (September-October 2007 for teal, September-October 2008 for tufted ducks).

### Experimental procedure

#### Disturbance

During the winter period, two groups of teal and one group of tufted ducks were disturbed over a one-week period. These disturbances were carried out three times at intervals of approximately 1.5 months (Table 1). Birds in group 1 (G1), teals only, were disturbed twice daily for 15 minutes between 08∶00 and 11∶00. In both species, birds in the group 2 (G2) were disturbed four times daily for 15 minutes during the same period of time. In each species, birds in the control group (CG) were not disturbed. During disturbance sessions, each aviary was monitored throughout the night via a night-view camera to ensure that the ducks were not disturbed by any other external factors.

**Table 1:** Date (mm/dd/yy) of the beginning and the end of the three disturbance sessions for each group in teals and tufted ducks.

		session 1		session 2		session 3	
		beginning	end	beginning	end	beginning	end
teals	G1	11/14/07	11/20/07	01/04/08	01/10/08	02/13/08	02/19/08
	G2	11/21/07	11/27/07	01/11/08	01/17/08	02/20/08	02/26/08
tufted ducks	G2	11/26/08	12/02/08	01/10/09	01/16/09	02/18/09	02/24/09

The disturbance was created by steering a radio-controlled car (E-Zilla FWD Hot-boddies™) towards the ducks at high speed until they took off. This was the most efficient way to induce simultaneous take-off flights for all birds in the group i.e. all birds flew from one end of the tunnel aviary to the other, hence leading to a response similar to that induced by a real predator. Furthermore, ducks had no previous experience of this type of stressor, therefore precluding any previous learning mechanism [29], [39]. No ducks were hurt by the car during these experiments. During disturbance phases, two experimenters (C.Z, M.B.) were near the aviaries to control the radio-controlled car and to record the number of individuals taking off.

#### Weighing and wing loading

In the two species, ducks in disturbed groups were caught with a net and were weighed (±1 g) in a nearby room the day before the beginning of each disturbance, on the fourth day and on the last day, immediately after the end of the last disturbance. Control birds were also caught and weighed at the same frequency as disturbed groups. The birds of each group were released together in their respective aviaries after weighing.

The wing area of the birds was determined from the outline of the stretched two wings, drawn onto paper. After the images were digitised, the wing area of each wing was measured using Sigma-Scan software (version 5.0). Total wing area was obtained by adding together the wing areas of the left and right wing of each individual. Wing loading (g.cm^-^^2^) was determined by dividing body mass by total wing area.

### Power Margin

Power margin was calculated from the power available (Pa) and the power requirement (Pr) for flight in all disturbed individuals before and after disturbance sessions. Equations for calculating Pa and Pr were derived from Norberg [40]. The equations are Pa = 21.94×m^(2/3)^ and Pr = 6.333×m^(7/6)^, where m denotes the body mass in kg and the power is in watts. The power margin (PM) is the ratio of the power available divided by the power required for flight (Pa/Pr). The PM gain is the difference between the PM values recorded before and after the disturbance.

### Food intake

Daily food intake determination began one week before each disturbance session and ended one week after its completion. Each day at 18∶00 the food remaining from the preceding 24 h was removed and food spilled on the tarpaulin was collected. One kg of standard duck fresh food (teal) and 0.8 kg of standard duck fresh food *plus* 0.8 kg of premium duck food (tufted duck) was then given to birds. The food given to the birds and removed from feeders was dried in an oven for 24 h at +40°C before being weighed to avoid errors due to changes in water content.

### Statistical analysis

Two-way repeated measures ANOVAs were used to test for differences in the number of individual daily flights between sessions and groups in teal, and also between sessions and sexes in tufted duck. We used general linear mixed models (GLMM) to examine the effects of disturbance on body mass and wing loading changes. Session, group, sex and state (before disturbance and after disturbance) were included as fixed factors. Session and state were also specified as repeated factors. Individuals were defined as a random factor to account for inter-individual variability. To examine the effect of disturbance on body mass loss, a GLMM was performed with session, group and sex as fixed factors. Session was specified as a repeated factor and individuals as random factor. For each disturbed group, we used a GLMM to test the difference in daily body mass loss between the first four days and the last three days of disturbance with session, sex and state (daily body mass loss during the first fourth days and daily body mass loss during the last third days). Session and state were used as repeated factors and individuals as a random factor. In teals, a GLMM was fitted to compare PM gain between the two disturbed groups. Session, group and sex were specified as fixed factors, session was also stated as a repeated factor and individuals as a random factor. In tufted ducks, the same model without the factor group was run to study PM gain in the disturbed group. For each GLMM, Tukey-Kramer multiple comparison adjustment was applied to obtain corrected p-values. Proc Mixed in SAS 9.1.3 (SAS Institute Corporation) was used to fit GLMM.

Daily food intake measurements have been investigated as time series. For each group and each session the autocorrelation functions (ACF) were calculated for all lags between −10 and 10. These functions give the correlation between the signal and itself with an increasing lag (i.e. correlation between the response at time t and the response at time t +l lag). This allowed us to investigate periodicity in the time series. In order to assess if a series is drawn at random, we used the Ljung-Box test. This is a portemanteau test which null hypothesis is “data are random” (the name portemanteau test refers to a test that is made for each lag). The tests were performed for each group and each session at each lag. Time series analyses were conducting using R 2.9.2. Probability levels <0.05 were considered as significant. Mean values are reported ± S.E.

## Results

In both species, escape flights, body mass, wing loading and body mass loss did not differ between sexes **(** Table 2, 3).

**Table 2:** Statistics values and P values for all simple fixed effects of each model and for useful interactions between fixed effects and multiple comparisons in teals.

	Parameter	Test value	P value
flight number	session	F_2,41_ = 4.3	0.04
	group	F_1,41_ = 163.7	<0.001
	session*group	F_2,41_ = 13.8	<0.001
	G2 session1 vs G2 session2	t_12_ = −0.5	0.99
	G2 session1 vs G2 session3	t_12_ = −4.3	0.005
	G2 session2 vs G2 session3	t_12_ = −3.8	0.01
body mass	session	F_2,46.5_ = 74.5	<0.0001
	group	F_2,23.8_ = 4.0	0.033
	sex	F_1,35.7_ = 0.44	0.51
	state	F_1,34.5_ = 538.5	<0.0001
	group*state	F_2,34.5_ = 41.2	<0.0001
	session*group*state	F_4,51.8_ = 25.6	<0.0001
	CG initial vs CG final	t_34.4_ = 6.4	<0.0001
	G1 initial vs G1 final	t_34.4_ = −14.9	<0.0001
	G2 initial vs G2 final	t_34.9_ = 18.9	<0.0001
wing loading	session	F_2,45.7_ = 55.7	<0.0001
	group	F_2,26.1_ = 4.1	0.028
	sex	F_1,39.4_ = 0.3	0.56
	state	F_1,33.1_ = 459.8	<0.0001
	session*group*state	F_4,51.4_ = 20.4	<0.0001
	CG initial vs G1 initial	t_30.6_ = 1.3	0.81
	CG initial vs G2 initial	t_31.1_ = 1.2	0.84
	G1 initial vs G2 initial	t_31.9_ = 0.0	1
	CG final vs G1 final	t_24.2_ = 3.4	0.02
	CG final vs G2 final	t_25.1_ = 4.3	0.002
	G1 final vs G2 final	t_25.3_ = −1.1	0.89
	G1 final session1 vs G2 final session 1	t_31.4_ = 0.9	1
	G1 final session2 vs G2 final session 2	t_34.5_ = −1.0	0.99
	G1 final session3 vs G2 final session 3	t_25.2_ = −1.2	0.99
body mass loss	session	F_2,34.8_ = 89.2	<0.0001
	group	F_2,19.9_ = 57.7	<0.0001
	sex	F_1,23.8_ = 0.9	0.36
	session*group	F_4,40.9_ = 22.7	<0.0001
	session1 vs session2	t_35.4_ = −6.8	<0.0001
	session1 vs session3	t_35.4_ = −12.9	<0.0001
	session2 vs session3	t_36_ = −8.0	<0.0001
	CG vs G1	t_17.6_ = 8.6	<0.0001
	CG vs G2	t_17.5_ = 8.5	<0.0001
	G1 vs G2	t_19_ = 2.6	0.049
power margin gain	session	F_2,22.9_ = 66.7	<0.0001
	group	F_1,20.5_ = 7.1	0.015
	sex	F_1,20.5_ = 0.0	0.99
	session*group	F_2,22.9_ = 3.63	0.043
	session1 vs session2	t_23.4_ = 9.7	<0.0001
	session1 vs session3	t_23.6_ = 11.7	<0.0001
	session2 vs session3	t_24_ = 4.7	0.0003
	G1 session1 vs G2 session1	t_24_ = −3.3	0.04
	G1 session2 vs G2 session2	t_23.1_ = −2.1	0.35
	G1 session3 vs G2 session3	t_21.5_ = −0.9	0.95

**Table 3:** Statistics values and P values for all simple fixed effects of each model and for useful interactions between fixed effects and multiple comparisons in tufted ducks.

	Parameter	Test value	P value
flight number	session	F_2,41_ = 118.1	<0.001
	sex	F_1,41_ = 3.8	0.10
	session*sex	F_2,41_ = 2.1	0.17
	session1 vs session2	t_23_ = −11.5	<0.0001
	session1 vs session3	t_23_ = −14.6	<0.0001
	session2 vs session3	t_23_ = −3.1	0.006
body mass	session	F_2,28.5_ = 47.2	<0.0001
	group	F_1,26.5_ = 28.2	<0.0001
	sex	F_1,26.5_ = 5.3	0.03
	state	F_1,23.3_ = 136.3	<0.0001
	session*group*state	F_2,30.2_ = 30.2	0.0009
wing loading	session	F_2,27.5_ = 47.0	<0.0001
	group	F_1,28.5_ = 18.3	0.0002
	sex	F_1,28.5_ = 0.3	0.60
	state	F_1,22.1_ = 115.8	<0.0001
	session*group*state	F_2,28.6_ = 8.9	0.001
	GC initial vs G2 initial	t_40.5_ = 1.3	0.55
	GC final vs G2 final	t_27.3_ = 6.8	<0.0001
	G2 final session1 vs G2 final session2	t_31.1_ = 1.2	0.98
	G2 final session1 vs G2 final session3	t_39.4_ = 2.6	0.30
	G2 final session2 vs G2 final session3	t_29.8_ = 1.7	0.86
body mass loss	session	F_2,23_ = 2.4	0.11
	group	F_1,15.7_ = 41.7	<0.0001
	sex	1.341.7	0.28
	session*group	F_2,23_ = 12.0	0.0003
	CG session1 vs CG session2	t_24_ = 1.8	0.51
	CG session1 vs CG session3	t_24_ = 2.0	0.37
	CG session2 vs CG session3	t_24_ = −0.4	0.99
	G2 session1 vs G2 session2	t_24_ = −3.0	0.07
	G2 session1 vs G2 session3	t_24_ = −5.1	0.0005
	G2 session2 vs G2 session3	t_24_ = −0.9	0.94
power margin gain	session	F_2,11_ = 4.0	0.048
	sex	F_1,12_ = 0.01	0.92
	session*sex	F_2,11_ = 1.5	0.26
	session1 vs session2	t_12_ = 2.0	0.16
	session1 vs session3	t_12_ = 3.0	0.03
	session2 vs session3	t_12_ = 0.3	0.97

### Teal

**Figure 1:**
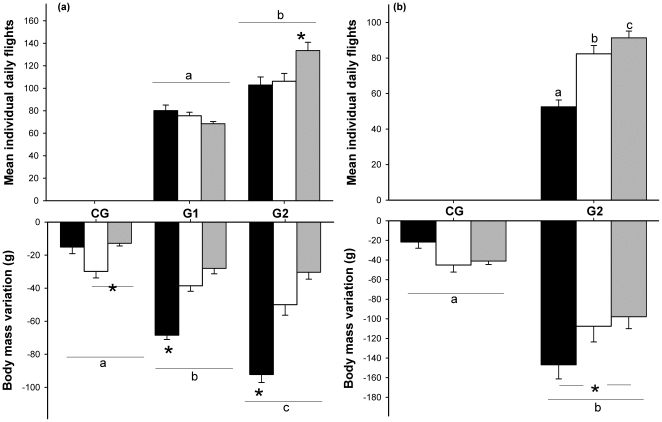
Escape flights and body mass variations. Mean number (± SE) of daily escape flights for the three sessions in disturbed groups and average body mass variations (g) for the three disturbance sessions in control and in disturbed groups in teals (a) and tufted ducks (b). In each group, black bars correspond to the first session, white bars to the second session and grey bars to the third session. Letters indicate significant difference between groups. * indicate differences between sessions in the different groups.

On average, G2 birds performed 1.5-fold more escape flights than those of G1 (Figure 1a). Individual daily flights did not differ between sessions in G1birds (*P*>0.07) but did in those from G2, with more flights during session 3 (Table 2, Figure 1a).

Body mass, wing loading and body mass loss differed over time between birds in the different groups (Table 2).

During disturbance, body mass loss was significant in birds in all three groups and differed significantly between the three sessions, with a greater loss in the first session (Table 2, Figure 1a). Birds in both treatment groups showed body mass loss at least two times higher than that seen in control birds (Table 4), and body mass loss was higher in G2 than in G1 (Table 2). Birds in disturbed groups showed a mean daily body mass loss that was not linear over the sessions, it was higher during the first four days of disturbance (G1 = −8.04±0.62 g.day^−1^, G2 = −10.38±0.83 g.day^−1^) than during the three last days (G1 = −2.77±0.45 g.day^−1^, G2 = −3.55±0.74 g.day^−1^) (Table 2).

**Table 4:** Mean (±SE) body mass loss (g), initial and final wing loading (g.cm^−^^2^) for each group and mean (±SE) power margin gain and relative power margin gain for the disturbed groups in the two species for all sessions.

		body mass loss (g)	initial wing loading (g.cm^−^^2^)	final wing loading (g.cm^−^^2^)	power margin gain	power margin gain (%)
	G1	45.0±2.4	0.90±0.02	0.78±0.02	0.43±0.03	7.3±0.4
teals	G2	57.3±4.1	0.90±0.02	0.75±0.02	0.57±0.04	9.6±0.7
	CG	19.3±1.7	0.94±0.03	0.89±0.03	/	/
tuftedducks	G2	117.4±8.8	1.43±0.02	1.19±0.01	0.39±0.03	9.6±0.7
	CG	36.0±3.7	1.48±0.01	1.41±0.01	/	/

Although initial wing loading did not differ between groups, final wing loading was lower in disturbed birds (G1 and G2) than control birds at the end of the disturbance sessions (Table 2, 4). For all sessions, final wing loading was similar in both disturbed groups (Table 2, 4).

**Figure 2:**
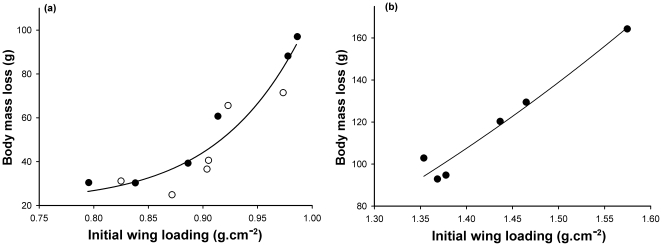
Body mass loss and initial wing loading. Relationship between body mass loss (g) and initial wing loading (g.cm^−^^2^) for the three disturbance sessions in disturbed groups in teals (a) and tufted ducks (b) (values plotted are means). In teals, individuals in G1 and G2 are indicated by open and closed circles, respectively. The relationship is best described by y = 85.31×^12.86^+21.92, R^2^ = 0.88, F_2,11_ = 37.36, p<0.0001 in teals and by y = 58.68×^2.77^−41.65, R^2^ = 0.93, F_2,5_ = 34.73, p = 0.008 in tufted ducks. The results indicate that body mass loss was higher when the initial wing loading was high.

Body mass loss was not related to final wing loading (*R^2^* = 0.09, *F*_2,11_ = 1.54, *P* = 0.27), but the greater the initial wing loading, the greater the body mass loss was seen to be (*R^2^* = 0.88, *F*_2,11_ = 37.36, *P*<0.0001, Figure 2a).

Power margin gain was higher during the first session (0.76±0.03) then decreased over the following sessions (session 2: 0.43±0.03 and session 3: 0.31±0.03). Power margin gain was greater in G2 than G1 birds, although the difference was only significant for the first session (G1: 0.65±0.04; G2: 0.87±0.05; Table 2).

**Figure 3:**
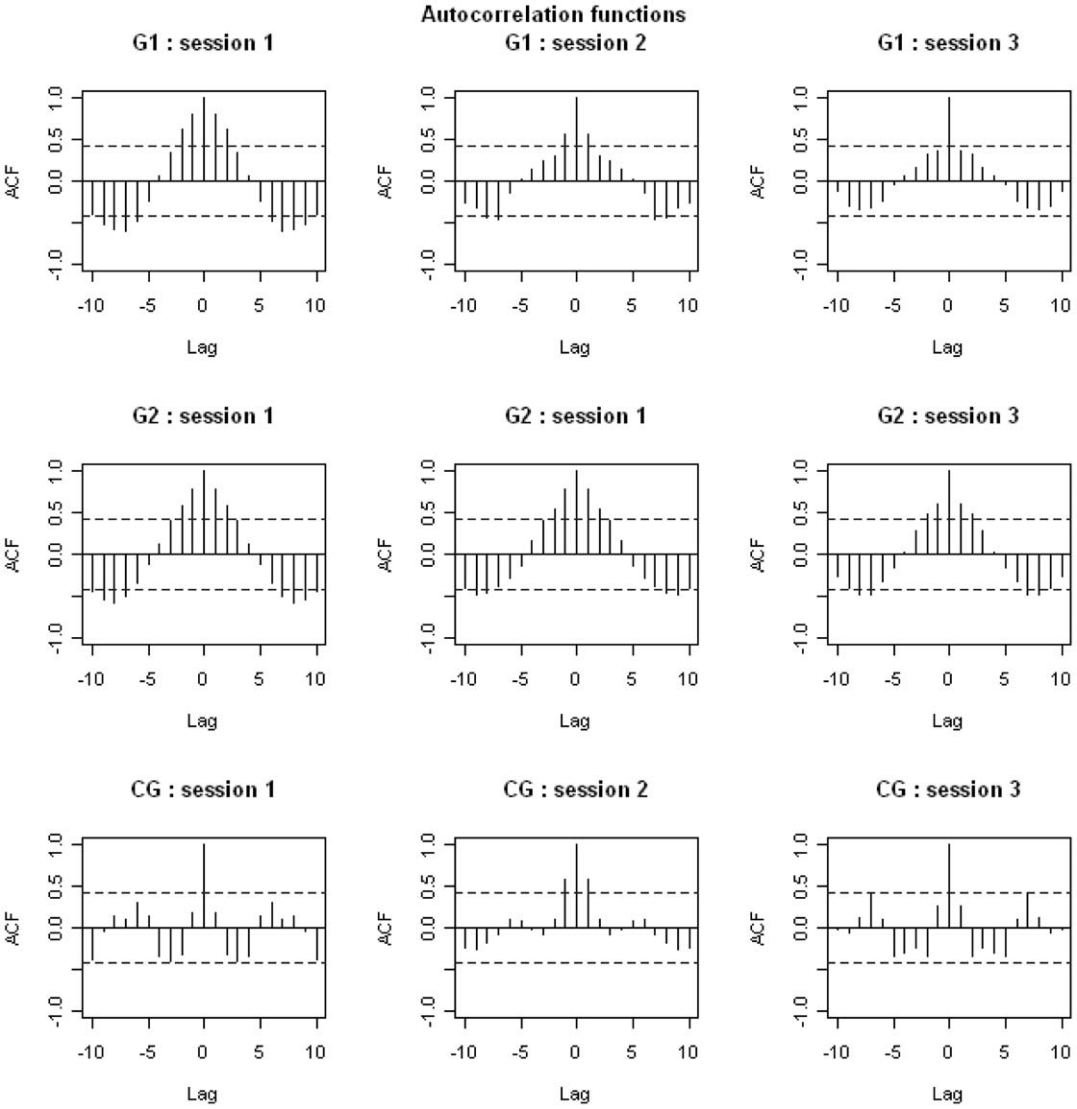
Autocorrelation functions relative to food intake in teals. Data are for each session, in the three groups. Dashed-lines represent the limit of the significant differences from 0 for the correlations. For example, for positive correlations, each correlation located above the dashed line is significantly different from 0 whereas each correlation located below the dashed-line is not significantly different from 0.

**Figure 4:**
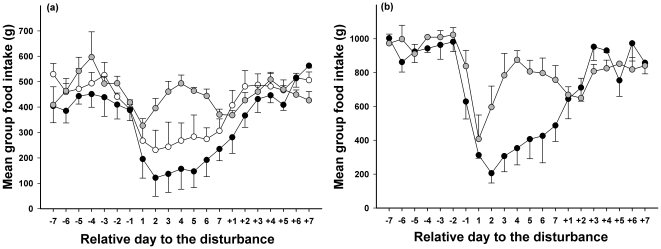
Mean food intake compared to average intake on the first day of disturbance. Panel a: teals, panel b: tufted ducks. Data were the mean value ± SE for the three sessions in control group (grey circles), group 2 (black circles) and group 1 (white circles).1 corresponded to the first day of disturbance.

The food intake of control birds was random, that is to say not dependent on previous food consumption (Figure 3). The food intake for birds in the disturbed groups was dependent on their food consumption over the 2 previous days. These results were confirmed by Ljung-box test (see figure S1). When comparing the food intake of a particular day with that recorded the same day of the previous or following week, correlations were stronger in G2 than in G1 birds (Figure 3). All correlations were negative, meaning that when the food intake was high one day it would be low 7 days later, and vice versa. This means that food intake decreased during the disturbance and returned to normal values following the disturbance; this decrease was greater in G2 than in G1 birds (Figure 4a).

### Tufted duck

The number of daily escape flights in the disturbed birds increased throughout the three sessions (Table 3, Figure 1b). Body mass, wing loading changes and body mass loss were different over time between disturbed and control birds (Table 3).

Body mass significantly decreased during the disturbance session in both groups, but this decrease was three times greater in disturbed birds than in control birds (Table 3, 4). Body mass loss was greater in the first than in the third session in disturbed birds, whereas it did not differ between sessions in control birds (Table 3, Figure 1b). Mean daily body mass loss in G2 birds was greater during the first four days of disturbance (−25.48±1.24 g.day^−1^) than during the last three days (2.06±1.74 g.day^−1^) (Table 3).

Initial wing loading did not differ between groups (Table 3, 4). Final wing loading at the end of disturbance in G2 birds did not differ between sessions and was significantly lower than in control birds (Table 3, 4).

Although, there was no relationship between final wing loading and body mass loss (*R^2^* = 0.75, *F*_2,5_ = 8.75, *P* = 0.06), we noted that the greater the initial wing loading was, the greater the body mass loss was seen to be (*R^2^* = 0.93, *F*_2,5_ = 34.80, *P* = 0.008, Figure 2b).

Disturbance led to a mean power margin gain of 9.6±0.7 % in G2. The power margin gain differed between sessions, values being higher in the first (0.47±0.04) and lower in the third (0.35±0.04) (Table 3).

**Figure 5:**
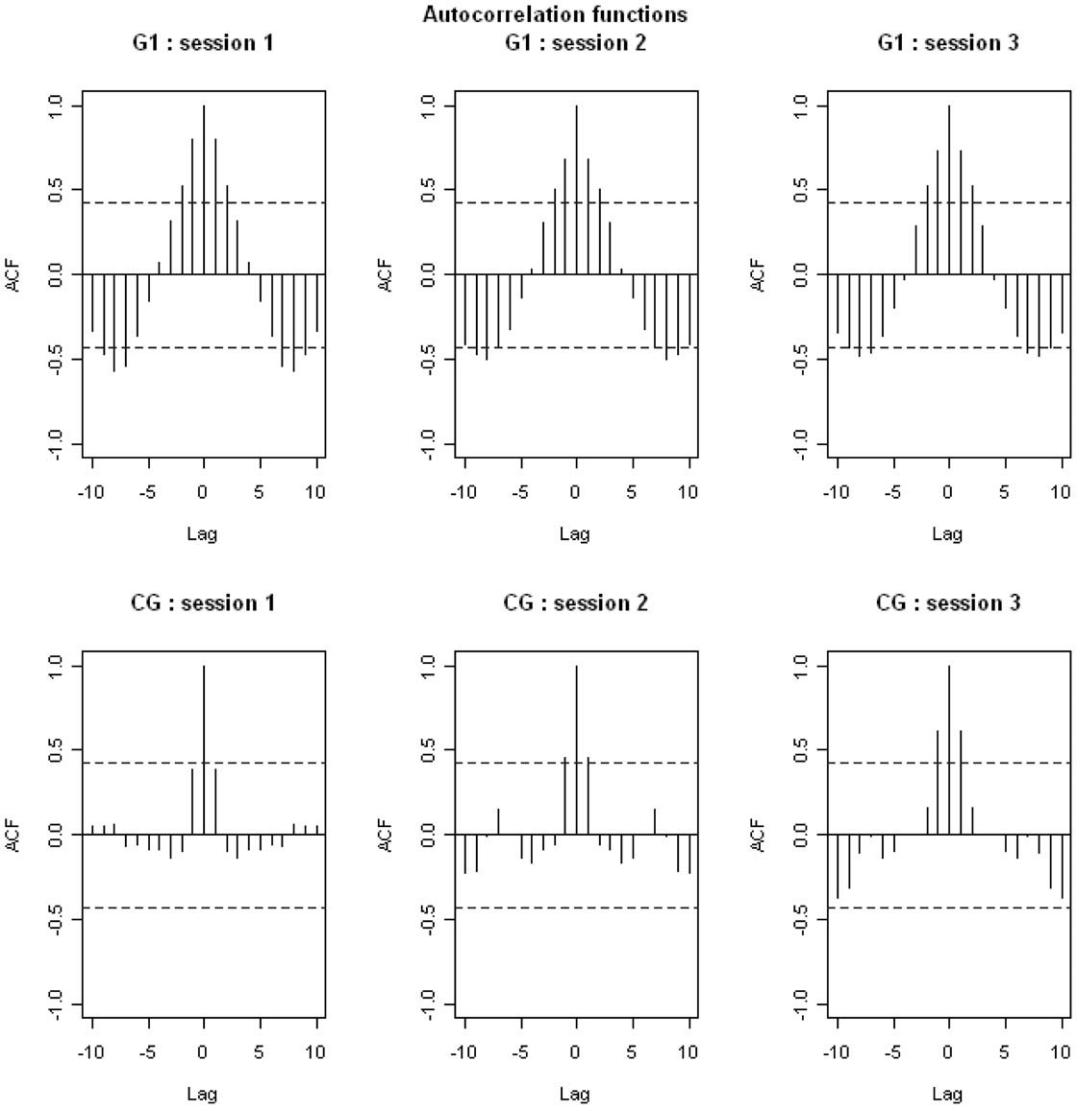
Autocorrelation functions relative to food intake in tufted ducks. Data are for each session, in both groups. Dashed-lines represent the limit of the significantly differences from 0 for the correlations. For example, for positive correlations, each correlation located above the dashed line is significantly different from 0 whereas each correlation located below the dashed line is not significantly different from 0.

Food intake depended on what had been eaten the previous day (Figure 5). In the disturbed group, food intake was dependent on that of the 2 previous days. These results were confirmed by Ljung-box test (see figure S2). The comparison of the food intake of a specific day with that recorded the same day of the previous or following week revealed negative correlations, indicating that if the food intake was high one day, it would be low 7 days later and vice versa. This means that food intake decreased during the disturbance and returned to its normal value after the disturbance ended (Figure 4b).

## Discussion

We show in this experimental study that teal and tufted ducks responded to an increased predation risk by reducing food consumption and body mass. This strategy is consistent with an improved capacity to escape, achieved by the reduction of wing loading. This study also confirms that the magnitude of the response to the starvation-predation risk trade-off depends on body reserve management strategy.

In both species, the relative body mass decrease was at least twice as high in the disturbed groups as in the control one. We suggest that this loss probably resulted from mass-dependent costs associated with disturbance. This result is in accordance with findings obtained in mallards and in passerines exposed to an increased predation risk [15], [16], [20]–[25], [29]. Such body mass decrease should be advantageous since it reduces energetic maintenance costs, foraging time and wing loading. In natural conditions, reducing the impact of these factors would allow reduced exposure to predators and enhanced escape capabilities [8], [9], [11], [18]. Our results therefore support the starvation-predation risk trade-off theory which predicts that an increase in predation pressure leads to a decrease in body mass [6], [9], [10]. Similarly, Nebel and Ydenberg [41] have shown that wing loading differences of non-breeding waterbirds are linked to changing predation risk.

Teal and tufted ducks in control groups lost a significant amount of weight, probably as a direct consequence of handling stress [3], [23], [42], [43]. Moreover, body mass loss was also higher in disturbed groups of both species during the first session than during the following ones. This could be related to a stress response associated with the novelty of the disturbance situation. During the first session, stress response associated with the mass-dependent costs of the disturbance may result in a greater body mass loss. However, this is unlikely to be the correct explanation, since corticosterone levels were the same in both disturbed and control groups, and were not seen to be higher during the first session in either species [Zimmer unpublished data]. Another possibility is that both species started the first session with relatively high fat stores, meaning that mass-dependent costs of disturbance flights were greater during this first session.

In tufted ducks, body mass loss was at its highest during the first session, during which the lowest number of daily escape flights was also recorded. Interestingly, the converse was observed during the third session (Figure 1b). A similar result was found in teal from the G2 disturbed group (Figure 1a). Escape flights require at least three times more energy expenditure than sustained flapping flights [44], [45]. Thus, if body mass loss is directly related to an increase in energy expenditure in response to the number of escape flights, one would expect a greater decrease in body mass to result from a larger number of flights. Our results do not support this prediction, therefore underlining the fact that the extent of body mass loss in disturbed groups for these two species is not consistent with an increase in the energy expenditure associated with escape flights.

Although food was provided *ad libitum*, food intake decreased during the week of disturbance in the treatment groups of both species (Figure 4). The food intake of disturbed groups was not random, but depended at least on food consumption of the previous day. Therefore, teals and tufted ducks did not increase their energy intake to compensate for the increased energy expenditure incurred by take-off flights. Both species are mainly nocturnal foragers whose diurnal foraging represents only approximately 10% of their total daily time budget [46]–[49]. Since the maximum disturbance in the study lasted for a maximum of 1 hour, (i.e. less than 12% of the daytime) we assume that ducks were not constrained by their available foraging time. The decrease in food intake does not fit the interrupted foraging theory, which predicts that birds should gain weight as a compensation mechanism in response to a reduced probability of feeding when predation risk rises [50], [51]. In teals, the dynamic of food consumption was the same in both disturbed groups despite pronounced body mass loss in G2. Consequently, the decrease in food intake should result in decreased body mass in order to gain efficiency in escape flights [19], [23], [29]. Improvement of flight capabilities could be achieved by adjusting the mass of different organ groups such as digestive compartments, pectoral muscle or fuel reserves [9], [10], [52], [53]. Nevertheless, it is unlikely that the weight loss in teals and tufted ducks could be explained solely by a reduction in the digestive compartment, since the birds never fasted. However, it would be interesting to check whether variation of pectoral muscle size could be decoupled from body mass variations [53]. As suggested by Van den Hout and colleagues [54] for other waterbirds, variation in pectoral muscle size could be due to the predator-escape tactic and should be smaller in gregarious species than in solitary-living species. Solitary-living species are particularly vulnerable to surprise attacks, and in this case rely on a speed-based escape that is made easier by increasing pectoral muscle size. In contrast, gregarious species can detect predators earlier and prepare themselves for an escape response facilitated by a decrease in body mass [54]. The gregarious behaviour of both species in this study [55] leads us to assume that muscle size is likely to remain relatively stable. Therefore, it seems that the body mass loss observed here was rather the result of a decrease in body reserves, but this can only be asserted after detailed studies on body composition variation during disturbance.

Flight mechanics theory and experimental studies show that a high body mass and associated high wing loading can impair flight capabilities during predator attacks by decreasing the speed and the angle at take-off and aerial manoeuvrability [10], [12]–[15], [17], [56], [57]. Indeed, wing loading is a major issue for flight performance and is mainly negatively related to flight speed [40]. Interestingly, we showed that body mass loss was positively linked to initial wing loading and not to final wing loading (Figure 2). In teals, although body mass loss differed between disturbed groups and sessions, final wing loadings never differed, and were lower than in control group birds. In tufted ducks, similar results have been obtained. It therefore appears that ducks under disturbance attempt to reach a target wing loading by adjusting body mass through the control of food intake. Furthermore, final wing loading did not differ among groups and sessions despite the different number of take-off flights. Thus, these results do not support the idea that physical training through repeated flights would have a major impact on flight performance, but rather suggest that wing loading at the beginning of an increased risk period is the main factor driving body mass regulation. This conclusion is also supported by the fact that body mass loss reached its highest values during the first part (1–4 days) of each disturbance session. Overall, disturbed ducks increased their power margin by 7 to 10 % during disturbance sessions, resulting in a higher availability of energy for flying. Birds hence have better manoeuvrability and can climb more easily [27], which probably also explains why they achieved more take-off flights throughout the disturbance session as body mass decreased. Overall, these results concord with those obtained for the mallard [29] and support the argument for an optimal adjustment of body mass and flight performance among different duck species in response to an increase in predation risk. It seems that these are strategic adjustments rather than an environmentally-induced response, since we observed the same adjustments in three different duck species with different body size and ecology. Environmental conditions, and particularly very harsh winter conditions, may affect response to predation and starvation risks and thus have an impact on energy reserve levels and body masses [58]. The response was nevertheless similar in all three species, particularly for the final wing loading in a given species during different periods in winter. Moreover, none of the three species of ducks encountered similar wintering ambient weather conditions. Our results are therefore in accordance with the starvation-predation risk trade-off theory [6], [7], suggesting that this is a general mechanism driving body mass and wing loading changes under different predation risks (see also [41]). It should be noted that results obtained when stressing a group of birds may differ from those observed in individually stressed birds. Metcalfe and Ure [17] pooled data of a group of alarmed zebra finches and showed that daily body mass change influences flight performances. Yet it has been shown in the same species that mass has little or no effect on flight velocity within the natural body mass range when birds were individually stressed [59]. Nevertheless, despite the fact that we stressed groups of birds in our study, all individuals were subjected to the same level of disturbance since ducks responded to the danger represented by the rapid approach of the car and it seems that ducks did not react to each other since some ducks can take-off before the rest of the group while some others can delay their take-off. Moreover, all disturbed ducks decreased their body mass to reach approximately equivalent wing loading in response to the disturbance. Finally, as ducks generally live in flocks in the wild, disturbing a flock of ducks closely recreates their behaviour in the wild.

**Figure 6:**
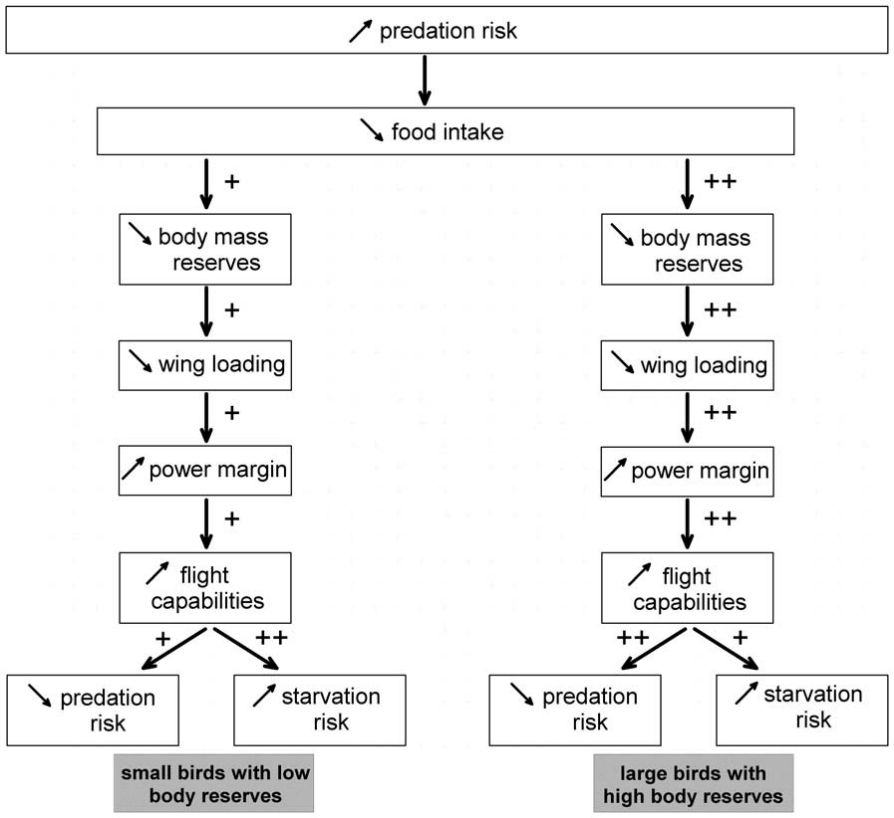
Hypothetical responses to an increase of predation risk. The diagram considers different types of bird species according to their theoretical amount of body reserves.

According to the results obtained in this study, the overall response to the aforementioned trade-off in a representative set of Anatidae species (tufted ducks and common teals, this study; mallards, [29]) is broadly similar in all three species in so far that it relates to body mass and food intake regulation. However, the study of large birds with high body reserves indicated a greater response to an increased predation risk compared to small birds with low body reserves, indicated by a body mass loss approximately two times higher in the former (6-16%) than in the latter (2-5%, [19], [22], [23]). In such large animals, the power margin is relatively low because the power available for flight increases with body mass at a slower rate than the required power [27]. As a result, high body mass loss leads to significant gains in power margin, which should be a key element for increased flight manoeuvrability during predator attacks in these ducks species. However, in birds laying down significant amounts of body reserves, predation risk is minimized without dramatically increasing starvation risk. Actually, the body mass at the end of each disturbance session was on average 50% higher than the values recorded in lean or depleted teals and tufted ducks ([60], Boos unpublished data), thus leading us to the conclusion that predation risks could be of greater importance in the regulation of body mass than the starvation risk. In contrast, in small birds like passerines, the achievement of the trade-off between both risks is quite different and, as shown in several studies, body mass loss in response to enhanced predation risk is more limited [19]–[25]. In fact as passerines store limited amounts of body reserves that only allow short-term resistance to fasting [4], [30] the risk of starvation is higher than predation risk. This phenomenon is amplified by their high specific metabolism, due to their small size and to the high proportion of lean mass which is energetically more costly to maintain than fat mass [34]. Furthermore, in passerines the power margin and consequently the manoeuvrability are very high as compared with larger ducks, and body mass reductions are of minor impact on these parameters (see [59], [61]–[63]). In contrast, it has been shown that in small migrant birds that build up relatively large fuel reserves before migration, this increase in body reserves (up to 67% of lean body mass) results in a reduction in the take-off angle and/or velocity, leading to a higher predation risk [12], [14], [64], [65]. During this period in small birds there is an increase in predation risk and a decrease in that of starvation. This situation could be comparable to that seen in birds with high body reserves. This study also shows that the response to the starvation-predation risk trade-off is broadly the same in a small and a medium size duck species as in a large one that manages body reserves in the same way. Therefore, we suggest that the relative importance of starvation and predation and the response to the trade-off between these risks are not directly related to the size of the species as proposed by Zimmer et al. [29] but are rather dependent on the strategy of body reserve management (Figure 6).

To conclude, this study revealed that an increase in predation risk leads to a strategic body mass loss in the teal and the tufted duck, most probably in order to reach a more favourable wing loading. This result echoes previous results obtained in the mallard [29] and with those observed in passerines [19], [21], [23]. Thus, the starvation-predation risk trade-off seems to be one of the major life history traits underlying optimal body mass adjustment in a variety of duck species, and we expect that these findings can be generalized to all birds facing disturbance or predation events. However, the relationship between the power margin gain and the decrease in body mass differed between duck species and passerines, probably because of different strategies in body reserve management. To confirm these results it will be necessary to specifically measure the impact of wing loading adjustment on flight performance and body fuel amount. Overall, body mass decline under disturbance is not implicitly deleterious when birds, depending on their body fuel reserves and metabolism changes, escape more efficiently and thus avoid immediate mortality by being caught. Finally, as shown in shorebirds [41], the benefits of wing loading adjustments and associated behaviour changes should be better addressed when dealing with the impact of disturbance on the fitness of birds, especially among waterfowl species.

## Supporting Information

Figure S1 **Ljung-Box test for food intake in teal.** In order to access if the data are random, we performed a Ljung-Box test that is a portemanteau test whose null hypothesis is “data are random”. (The name portemanteau test refers to a test that is made for each lag.) The tests were performed for each group of teal and each session at each lag. The p-values are drawn in the graphic to have a better sight on the results. In the control group, for each session and almost all lags, the null hypothesis can not be rejected so that we can consider that this data are drawn at random. The graphics for the group 2 are clearly showing really low p-value so that the data are not drawn at random so there is a pattern in the data. It is almost the same as regards group 1 with an exception for session 3 where most of the p-values are larger than 0.05. However, this has to be seen in parallel of the ACF for this session. As a matter of fact none of the correlations are significant but the shape of the ACF is typical of periodic (so not random) process.
(TIF)

Figure S2 **Ljung-Box test for food intake in tufted duck.** In order to access if the data are random, we performed a Ljung-Box test. The tests were performed for each group of tufted ducks and each session at each lag. The p-values are drawn in the graphic to have a better sight on the results. In the control group at session 1, for all lags, the null hypothesis can not be rejected so that we can consider that this data are drawn at random. At session 2, only the p-value related to lag 1 is lower than 0.05. Thus, except for the food intake of the next day the data are random. At session 3, p-values related to lag 1 to 3 and from 10 to 14 are lower than 0.05. Indeed, even if the data are not totally random, there is still a lot of randomness in the drawing of the data. The graphics for the disturbed group are clearly showing really low p-value so that the data are not drawn at random so there is a pattern in the data.
(TIF)
